# A Case of Compound Comminuted Fracture of the Humerus, and Simple Fracture of the Tibia and Fibula—Right Arm and Leg

**Published:** 1865-09

**Authors:** E. J. Nugent

**Affiliations:** Homer, Ill.


### A CASE OF COMPOUND COMMINUTED FRACTURE OF THE HUMERUS, AND SIMPLE FRACTURE OF THE TIBIA AND FIBULA—RIGHT ARM AND LEG.

By E. J. NUGENT, M. D., of Homer, Ill.

On Saturday, 3d of December, 1864, I was sent for in great haste to go to Mr. John Insley's, five miles south-west ot this place. On my arrival at the place, I found his son, a boy of about ten years of age, lying upon the bed, very badly bruised and wounded. Upon inquiry in regard to the facts, I learned that they were engaged in threshing, it being a very cold windy day, his son having on a rather long overcoat, was standing not far from the tumbling rod of the machine, and the wind blowing, his coat was caught by the rod and he drawn by the force of its revolutions until he was carried, as they could best judge, some twelve or fifteen times in its revolutions around, when one of the men, seeing his condition, caught the rod, and as fortune would have it, one of the rivets being loose, it was readily displaced and probably the boy's life saved, before the horse-power could be stopped. The boy was carried to the house almost insensible.

I proceeded to adjust and place in position the fractured tibia and fibula by means of bandage, pasteboard splinters, roller, etc., and placed the whole in a neat fracture-box, made especially for the occasion.

There was a wound of the right *arm*, commencing about the inner portion of the coraco-brachialis, involving the integuments and superficial fascia and extending laterly and posteriorly through all of the muscles and soft parts of the arm, ranging posteriorly about midway between the spine of the scapula and his doral vertebra—deep down through the tropezius, and the lower portion of the arm was twisted around, but no wound of the brachial artery or its sheath. The fracture was at about the surgical neck of the humerus, and but slight hemorrage, the fractured ends of the bone protruded and pierced through the shirt, exhibiting the nature of the fracture.

After stating to his father the serious nature of his wounds, I requested consultation. He would not listen to resection or amputation, but was exceedingly anxious to save the arm.

A messenger was immediately sent for my venerable friend, Dr. Somers, of Urbana, who did not arrive until four o'clock A. M., Sabbath morning. Dr. W. A. Conkey, of our place, also accompanied me in the morning.

10, A. M.—Pulse 80. Dr. W. A. Conkey then proceeded to administer chloroform. About one hour was spent in striving to place him under its influence, but all to no effect. Dr. Somers purchased the chloroform, said it was fresh and good as usual, of common strength. We then proceeded without it. I applied some 10 or 15 interrupted sutures and adhesive plasters, in strips, and brought the wound nicely, and close in opposition, and then produced extension and applied roller and four well padded splinters, and secured the whole by bandages, and placed a wedge-shaped pad in the axilla, and extended the arm and placed it upon a pillow. The little fellow endured his suffering like a man, and rested from the effects of morphia very well afterwards. Left him six powders of sulph. morphia, ½ gr. each, to produce rest and allay pain, and ordered Epsom salts ℥i. in the morning.

Dec. 5.—Found my little patient had rested tolerably well last night, and the dose of salts being repeated without effect, an enema was administered, which produced rather a copious discharge; pulse 110; tongue coated light-yellowish, some appetite, had eaten some and drank milk; parts swelled considerable.

Dec. 6.—This morning the patient feels very well, having rested last night, had one operation from the bowels, appetite moderate, pulse 100.

Dec. 7.—To-day applied new bandages, and dressed the arm, wound is suppurating very nice, and looks well; took away some of the sutures and applied adhesive strips anew.

Dec. 8.—Visited him again to-day; he feels very comfortable; bowels regular; pulse 95; tongue clearing off from tip and edges; wound suppurating profusely.

Dec. 9.—Doing very well; rested well last night; appetite improving; ordered light farinaceous diet; milk for drink; dressed the wound; applied new adhesive strips; is suppurating a great deal, and but little tumefaction of the parts.

Dec. 12.—Visit him now every other day; dressed the wound; removed all the sutures; applied new adhesive strips and bandages; wound suppurating very much, and was compelled to change bandages, and pad the splints anew, and changed the bed-clothing, to keep from producing offensive smell.

Dec. 14.—Dressed the wound to day; suppurating nicely; parts look well; bowels regular; appetite good; rests well; tongue almost clean.

Dec. 16.—Dressed wound to-day; doing well and improving fast.

Dec. 18.—Dressed wound; applied all new dressings; wound commencing to unite; parts healthy.

Dec. 20.—Again I visited my little patient and dressed his wounds—all look healthy—suppurating and healing as fast as possible.

And without lengthening the report by naming every visit, I will conclude by adding that I continued to visit and dress the wound every second or third day, up to about the latter part of January, when I was able to place the limb in pasteboard splints and he could use the arm some, it having united firm and solid to all appearance. The external wound healed very nicely, but leaving, as it could not be prevented, a large cicatrix. The placing of this arm in splints was a great risk, but the father consented and urged us to do so, as he would stand for all damages that would occur; and contrary to the expectations of us all, he will have good use of the arm and leg, and a novelty in the line of surgery of the complete union of a compound comminuted fracture; but everything seemed in his favor—cold weather, a healthy, vigorous constitution, and good care and attention, and everything that would conduce to his comfort.

I send you this case, hoping that it may be of some advantage to those who may read it, as it has been to me. Drs. Somers and Conkey have my acknowledgments for their aid and assistance in this case.

The patient can now use his arm about as well as the one not broken, in extension and raising and throwing stones with it, etc.

